# Efficacy and safety of tacrolimus-based treatment for non-rapidly progressive IgA nephropathy

**DOI:** 10.3389/fphar.2023.1189608

**Published:** 2023-05-18

**Authors:** Lijuan Zhao, Yanyan Yang, Hao Xu, Wei Leng, Guoshuang Xu

**Affiliations:** ^1^ Department of Nephrology, Xijing Hospital, Fourth Military Medical University of PLA, Xi’an, China; ^2^ Department of Nephrology, Shaanxi University of Chinese Medicine, Xianyang, Shaanxi, China; ^3^ M.S. in Biostatistics, Columbia University Mailman School of Public Health, New York, NY, United States

**Keywords:** IgA nephropathy, tacrolimus, proteinuria, immunosuppressive agents, adverse events

## Abstract

In this study, we aimed to evaluate the efficacy and safety of tacrolimus-based treatment for immunoglobulin A nephropathy (IgAN). We retrospectively reviewed 127 adult patients with primary IgAN with 24 h urine total protein quantity (24 h UTP) ≥ 1 g and serum creatinine ≤3 mg/dL. All patients were divided into tacrolimus (TAC) and control (non-TAC) groups according to the treatment strategy. Proteinuria remission, remission rate, and adverse events were compared between the two groups. Among the 127 patients, 61 received TAC-based treatment and 66 received non-TAC treatment. TAC group exhibited a more rapid decline in proteinuria than the non-TAC group at 3, 9, and 12 months (*p* = 0.049, 0.001, and 0.018, respectively). Remission rates at 1, 3, 6, 9, and 12 months were 41.0, 68.9, 80.3, 90.2, and 88.5%, respectively, in the TAC group. These rates were higher than those in the control group at 3, 9, and 12 months (*p* = 0.030, 0.008, and 0.026, respectively). Complete remission rates at 1, 3, 6, 9, and 12 months were 6.56, 19.7, 37.7, 54.1, and 62.3%, respectively, in the TAC group. These rates were higher than those in the control group at 9 and 12 months (*p* = 0.013 and 0.008, respectively). The estimated mean time to complete remission was significantly shorter in the TAC group than in the control group (*p* = 0.028). TAC did not increase the incidence of adverse events. In conclusion, TAC accelerated proteinuria remission in patients with non-rapidly progressive IgAN with no increased risk of adverse events. Further prospective randomized controlled trials are necessary to validate our findings.

## Highlights

1. Tacrolimus increased proteinuria remission in patients with IgA nephropathy
2. Tacrolimus-based treatment resulted in a higher remission rate than non-tacrolimus treatment
3. Tacrolimus did not increase the incidence of adverse events

## 1 Introduction

Immunoglobulin A nephropathy (IgAN) is the most common form of glomerulonephritis that is characterized by prominent mesangial deposits of IgA (Rajasekaran et al., 2021). IgAN is the primary cause of end-stage renal disease worldwide, with up to 30%–50% of the affected patients developing end-stage renal disease within 20–30 years after diagnosis (Maixnerova & Tesar, 2020). Proteinuria, glomerular filtration rate, and hypertension have been reported as the independent indicators of poor outcomes in patients (Maixnerova et al., 2014; Tan et al., 2015). Increased neutrophil-to-lymphocyte ratio, serum uric acid levels, and deposition of complement C3 have also been identified as independent risk factors for the progression of IgAN (Caliskan et al., 2016; Wang et al., 2021). Patients with IgAN have various clinical presentations and histological lesions, resulting in highly variable outcomes. Clinical presentation of IgAN ranges from asymptomatic hematuria to rapidly progressing renal failure (Pattrapornpisut et al., 2021). Prevalence of IgAN varies according to age. IgAN is most prevalent in Asians, with high risk of disease progression, severe clinical manifestations, and commonly reported active lesions, than in Europeans (Zhang & Barratt, 2021), indicating the necessity of using different therapeutic strategies for different patient populations.

To date, there is no established standardized treatment for IgAN owing to variations in the prevalence and progression of this disease. Treatments for IgAN focus predominantly on supportive care, such as measures to reduce proteinuria, decrease blood pressure, and minimize lifestyle risk factors, and non-specific immunosuppression (Floege et al., 2021). Rapidly progressive IgAN is defined as a ≥50% decline in eGFR over ≤3 months. Patients with rapidly progressive IgAN have a poor outcome and should be offered treatment with cyclophosphamide and glucocorticoids (Kidney Disease: Improving Global Outcomes KDIGO, 2021). Nevertheless, the additive benefits of currently available immunosuppressive agents, such as glucocorticoids (GCs), acetazolamide, cyclophosphamide, and mycophenolate mofetil (MMF), remain controversial (Floege et al., 2021; Pattrapornpisut et al., 2021; Aron, 2022), especially in non-rapidly progressive diseases (Natale et al., 2020).

Tacrolimus (TAC) is a calcineurin inhibitor widely used to treat autoimmune diseases and an anti-rejection drug for organ transplants (Ong & Gaston, 2021; Schumacher et al., 2021). TAC binds to T lymphocyte-specific FK506-binding protein (FKBP) to form the TAC-FKBP12 complex, which then binds to calcineurin and inhibits the expression of cytokines related to T-lymphocyte activation, thereby inhibiting the immune response (Kandikattu & Mishra, 2018; Ong & Gaston, 2021). TAC is also used for the immunosuppressive treatment of primary and secondary glomerular diseases (Hannah et al., 2016; Zhu et al., 2017), including IgAN. Several studies have demonstrated the efficacy of TAC in the treatment of refractory IgAN (Zhang et al., 2012; Hu et al., 2018; Yan et al., 2022). However, its efficacy and safety in patients with non-rapidly progressive IgAN remain unknown. In this study, we retrospectively reviewed 127 cases of non-rapidly progressive IgAN to evaluate the safety and efficacy of TAC-based treatment for IgAN by comparing it with non-TAC treatment.

## 2 Materials and methods

### 2.1 Patients

This retrospective cohort study enrolled patients with primary IgAN who underwent treatmet at the Xijing Hospital, Fourth Military Medical University, between August 2017 and July 2020. Inclusion criteria were as follows: 1) patients diagnosed with primary IgAN via renal biopsy and 2) decrease in the estimated glomerular filtration rate (eGFR) to <25% within 3 months. Exclusion criteria were as follows: 1) patient age <16 or ≥75 years, 2) 24 hurine total protein quantity (24 hUTP) < 1 g, 3) serum creatinine >3 mg/dL, 4) follow-up period <1 year, 5) presence of comorbidities, such as diabetes, hepatitis B, cancer, and amyloidosis, and 6) incomplete data or self-discontinuation of treatment. A total of 127 patients were enrolled in the study and divided into TAC-based (*n* = 61) and non-TAC (control, *n* = 66) treatment groups based on their therapeutic strategy. This study was approved by the Ethics Committee of Xijing Hospital, and the requirement for informed consent was waived because of the retrospective study design.

### 2.2 Treatment protocol

TAC was administered at a dose of 0.05–0.1 mg/kg/d, orally divided twice a day, and which was adjusted according to the blood concentration as 5–10 ng/L (Kidney Disease: Improving Global Outcomes KDIGO, 2021). TAC dose was gradually decreased (25%–33% decrease every 2 months) 3 months later or at the time of complete remission of proteinuria and was not changed when the dose decreased to 1 mg/d, and this treatment plan was maintained for 12 months. The treatment regimen in the non-TAC group included GCs, GC plus cyclophosphamide (GC + CTX), GC plus MMF (GC + MMF), and MMF. GCs were initially administered at dose of 30–50 mg/d with oral administration once a day, which was gradually decreased to 5 mg/month after 8 weeks and then to 2.5 mg/month when the dose tapered to 20 mg. CTX was administered intravenously at a dose of 0.8 g (weight ≤70 kg) or 1.0 g (weight >70 kg) per month and stopped when the total dose reached 7–8 g. MMF was administered at a dose of 1.5 g/day with oral administration twice a day, which was gradually decreased to 0.5 g/day 6 months later or at the time of complete remission of proteinuria, and this treatment plan was maintained for 12 months. Renin angiotensin system (RAS) inhibitors were used in all patients who tolerated RAS inhibitors, and the dose was gradually increased to the maximum tolerable dose over 3 months and maintained until the end of the study.

### 2.3 Data collection

Two authors (L.Z. and Y.Y.) simultaneously collected and entered the data, and any disagreements were resolved via discussion. Data, including sex, age, complications, renal biopsy, biochemical indices (blood routine, alanine aminotransferase, aspartate transaminase, cholesterol, triglyceride, blood glucose, uric acid, electrolyte, creatinine, eGFR, and 24hUTP) before treatment, and medications, were retrieved from the medical records of the patients. All patients were followed-up at least once every one-to-three months, and the above-mentioned biochemical indices and adverse events were recorded.

### 2.4 Outcomes and definitions

Complete remission (CR) was defined as urinary protein excretion <0.3 g/day with a decrease in eGFR to <10% of baseline. Partial remission (PR) was defined as a decrease in proteinuria to >50% of baseline and a decrease in eGFR to <10% of baseline. Patients with decreased proteinuria (<50% of baseline) or eGFR (>10% of baseline) were considered to be resistant to TAC-based treatment. Remission time was defined as the time from the start of TAC-based treatment until a complete or partial response was achieved. CR, PR and total response rates were calculated and recorded separately at months 1, 3, 6, 9, and 12.

### 2.5 Statistical analysis

Continuous and categorical variables were expressed as the means with standard deviations and event numbers with percentages, respectively. Normally distributed continuous variables were compared using the *t*-test, while others were subjected to the Mann–Whitney rank test. Categorical variables were analyzed using the Chi-square or Fisher’s exact tests. Statistical analysis was conducted using the SPSS 16.0 and GraphPad Prism 9.0 software packages. Statistical significance was set at *p* < 0.05. Estimated mean complete remission time was calculated using Kaplan–Meier curves and compared using the log-rank test.

## 3 Results

### 3.1 Baseline characteristics of enrolled patients

**FIGURE 1:**
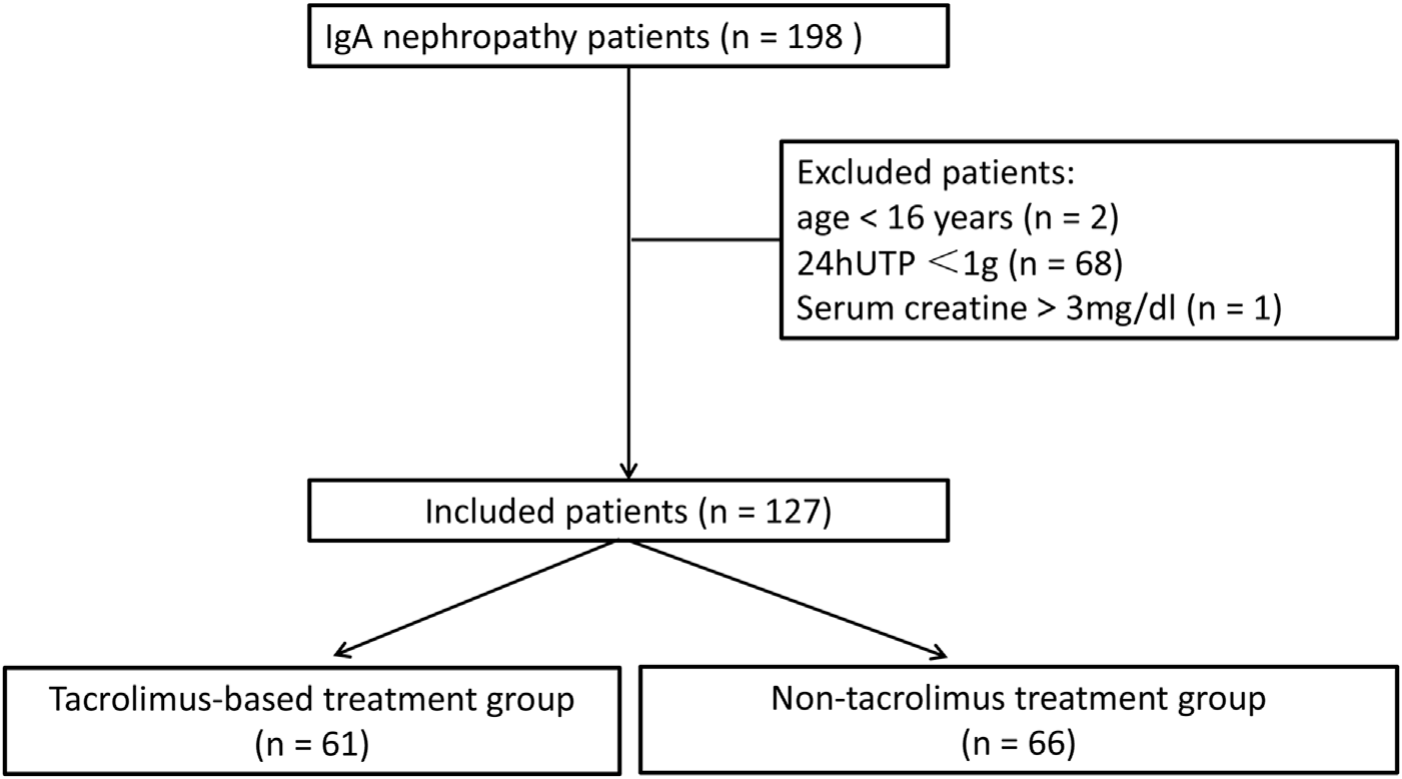
Selection process of patients included in this study.

Between August 2017 and July 2020, 198 patients were diagnosed with primary IgAN. Of these, 71 patients who did not meet the inclusion criteria, including two patients aged <16 years, 68 patients with 24 hUTP <1 g, and one patient with serum creatinine >3 mg/dL, were excluded from the study. Finally, 127 patients were enrolled, of whom 61 received TAC-based treatment and 66 received non-TAC treatment (Figure 1). Baseline characteristics of the included patients are presented in Table 1. The mean age of 127 patients was 40.6 ± 12.5 years, and 59.1% patients (*n* = 75) were males. Baseline serum albumin was 39.1 ± 6.8 g/L. Baseline creatinine and eGFR were 89.6 ± 35.3 μmol/L and 90.9 ± 30.0 mL/min/1.73 m^2^, respectively. Baseline 24 hUTP was 2,251.7 ± 1970.3 mg. Of the 127 patients, approximately 97.6% (*n* = 124) were treated with RAS blockade. All baseline characteristics showed no statistically significant differences between the two groups, indicating the comparability of the clinical outcomes of the treatments.

**TABLE 1:** Baseline characteristics of enrolled patients.

Variables	All (*n* = 127)	TAC group (*n* = 61)	Non-TAC group (*n* = 66)	*p*-value
Male gender (*n*, %)	75, 59.1%	33, 54.1%	42, 63.6%	0.286
Age (years)	40.6 ± 12.5	40.0 ± 13.1	41.2 ± 12.0	0.596
Hemoglobin (g/L)	139.5 ± 20.4	137.5 ± 18.4	141.3 ± 22.1	0.289
White blood count (10^9^/L)	8.1 ± 2.9	7.8 ± 2.7	8.3 ± 3.0	0.34
Platelet (10^9^/L)	240.3 ± 74.2	237.5 ± 68.1	242.9 ± 79.6	0.685
ALT (U/L)	22.3 ± 13.4	21.3 ± 9.5	23.2 ± 16.2	0.447
AST (U/L)	20.1 ± 10.5	20.4 ± 9.0	19.8 ± 11.8	0.746
Serum total bilirubin (mmol/L)	11.9 ± 5.7	11.1 ± 4.3	12.8 ± 6.6	0.092
Serum total protein (g/L)	66.3 ± 8.3	65.3 ± 9.2	67.2 ± 7.2	0.196
Serum albumin (g/L)	39.1 ± 6.8	37.9 ± 7.3	40.2 ± 6.0	0.062
Serum urea (mmol/L)	6.8 ± 4.8	6.6 ± 6.2	6.9 ± 3.0	0.758
Serum creatinine (umol/L)	89.6 ± 35.3	87.7 ± 37.4	91.50 ± 33.5	0.547
eGFR (ml/min/1.73 m^2^)	90.9 ± 30.0	93.5 ± 29.8	88.5 ± 30.2	0.354
Urine acid (umol/L)	356.2 ± 93.1	350.5 ± 88.9	361.4 ± 96.9	0.514
Urine protein (mg/24 h)	2,251.7 ± 1970.3	2,431.9 ± 2,123.8	2085.1 ± 1817.3	0.324
Use of RAS blocks (*n*, %)	124, 97.6%	60, 98.1%	64, 97.0%	0.54

TAC, tacrolimus; ALT, alanine aminotransferase; AST, aspartate transaminase; eGFR, estimated glomerular filtration rate; RAS, renin-angiotensin system.

### 3.2 Responses to treatment

#### 3.2.1 Changes in urine protein and serum albumin levels

**FIGURE 2:**
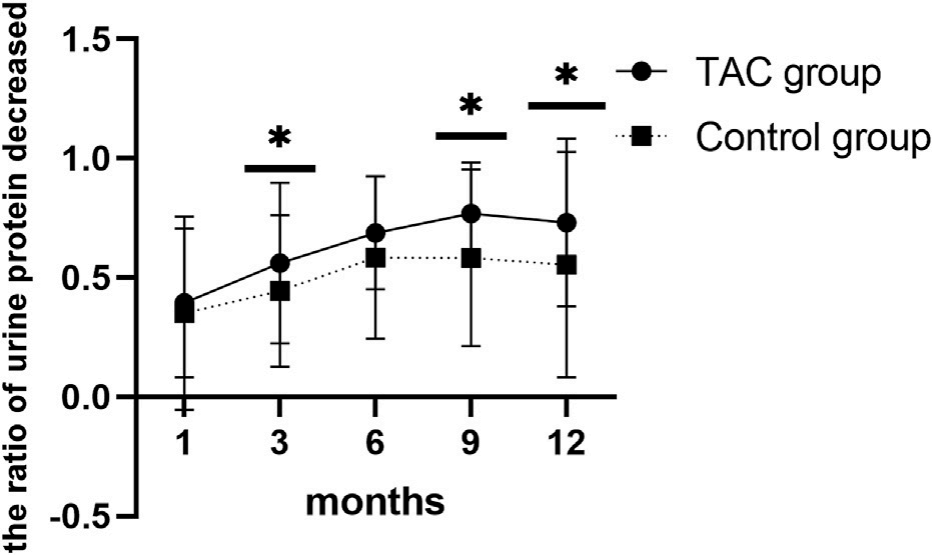
Rate of urinary protein decline during patient follow-up. TAC, tacrolimus.

Urine protein levels decreased significantly 1 month after TAC-based treatment (2,431.9 ± 2,123.8 vs. 1,515.6 ± 1,651.3; *p* = 0.009). Serum albumin levels also increased 1 month after TAC-based treatment, with no statistical significance (37.9 ± 7.3 vs. 39.9 ± 6.2; *p* = 0.106), and 3 months after TAC-based treatment (37.9 ± 7.3 vs. 42.6 ± 4.8; *p* < 0.0001). Urine protein levels decreased (2085.1 ± 1817.3 vs. 1,221.8 ± 1,074.0; *p* = 0.091) and serum albumin levels (40.2 ± 6.0 vs. 41.4 ± 4.2; *p* = 0.105) also increased 1 month after non-TAC treatment with no statistical significance. Changes in urine protein and serum albumin levels during the 12-month treatment plan in both groups are presentedin Table 2. Urine protein levels persistently decreased and serum albumin levels persistently increased with increasing treatment time in both groups. However, TAC group showed higher decrease in urine protein levels than the non-TAC group at 3 months (56.1 ± 33.7 vs. 44.5 ± 32.0; *p* = 0.049), 9 months (76.9 ± 21.4 vs. 58.2 ± 37.1; *p* = 0.001) and 12 months (73.2% ± 35.2% vs. 55.4% ± 47.2%; *p* = 0.018) (Figure 2).

**TABLE 2:** Changes of urine protein and serum albumin during the 12-month treatments.

	Time	TAC group (*n* = 61)	Non-TAC group (*n* = 66)	*p*-value
Urine protein (mg/24 h)	1-month	1,515.6 ± 1,651.3	1,221.8 ± 1,074.0	0.242
	3-month	960.4 ± 943.2	985.7 ± 687.0	0.862
	6-month	748.9 ± 884.2	729.9 ± 601.7	0.887
	9-month	550.8 ± 665.8	710.6 ± 603.6	0.159
	12-month	560.5 ± 832.3	700.3 ± 711.7	0.313
Ratio of urine protein decreased (%)	1-month	39.4% ± 31.2%	35.1% ± 40.5%	0.508
	3-month	56.1% ± 33.7%	44.5% ± 32.0%	0.049
	6-month	68.7% ± 23.7%	58.5% ± 34.0%	0.051
	9-month	76.9% ± 21.4%	58.2% ± 37.1%	0.001
	12-month	73.2% ± 35.2%	55.4% ± 47.2%	0.018
Serum albumin (g/L)	1-month	39.9 ± 6.2	41.4 ± 4.2	0.119
	3-month	42.6 ± 4.8	42.3 ± 4.5	0.714
	6-month	42.8 ± 5.5	43.2 ± 3.7	0.706
	9-month	43.31 ± 2.9	42.8 ± 3.8	0.397
	12-month	44.3 ± 2.9	43.5 ± 3.0	0.185
Ratio of serum albumin increased (%)	1-month	7.3% ± 16.8%	5.3% ± 19.7%	0.533
	3-month	16.1% ± 24.9%	8.2% ± 26.1%	0.082
	6-month	17.5% ± 29.3%	10.7% ± 25.3%	0.165
	9-month	20.2% ± 30.6%	9.4% ± 28.0%	0.058
	12-month	21.1% ± 31.3%	13.9% ± 32.8%	0.317

TAC, tacrolimus.

#### 3.2.2 Remission rate

As shown in Table 3, the remission rates at 1, 3, 6, 9, and 12 months after TAC-based treatment were 41.0, 68.9, 80.3, 90.2, and 88.5%, respectively. In the non-TAC group, the remission rates at 1, 3, 6, 9, and 12 months were 39.4, 48.5, 69.7, 71.2, and 71.2%, respectively. Moreover, the remission rate was 68.9% 3 months after TAC-based treatment, which was significantly higher than that in the non-TAC group (68.9% vs. 48.5%; *p* = 0.030). Similarly, the remission rates 9 months (90.2% vs. 71.2%; *p* = 0.008) and 12 months (88.5% vs. 71.2%; *p* = 0.026) after TAC-based treatment were significantly higher than those in the non-TAC group. After TAC-based treatment, CR rates at 9 months (54.1% vs. 31.8%, *p* = 0.013) and 12 months (62.3% vs. 37.9%, *p* = 0.008) were significantly higher than those in the non-TAC group. Overall, the TAC group showed a markedly higher total remission (95.1% vs. 83.3%; *p* = 0.047) and CR (62.3% vs. 42.4%; *p* = 0.033) rates than the non-TAC group.

**TABLE 3:** Remission rates of treatments in two groups.

Time		TAC group (*n* = 61)	Non-TAC group (*n* = 66)	*p*-value
One-month	R	25 (41.0%)	26 (39.4%)	0.859
	PR	21 (34.4%)	22 (33.3%)	1.000
	CR	4 (6.56%)	4 (6.1%)	1.000
3-months	R	42 (68.9%)	32 (48.5%)	0.030
	PR	30 (49.2%)	26 (39.4%)	0.288
	CR	12 (19.7%)	6 (9.1%)	0.126
6-months	R	49 (80.3%)	46 (69.7%)	0.220
	PR	26 (42.6%)	28 (42.4%)	1.000
	CR	23 (37.7%)	18 (27.3%)	0.256
9-months	R	55 (90.2%)	47 (71.2%)	0.008
	PR	22 (36.1%)	26 (39.4%)	0.718
	CR	33 (54.1%)	21 (31.8%)	0.013
12-months	R	54 (88.5%)	47 (71.2%)	0.026
	PR	16 (26.2%)	22 (33.3%)	0.440
	CR	38 (62.3%)	25 (37.9%)	0.008
Total	R	58 (95.1%)	55 (83.3%)	0.047
	PR	20 (32.8%)	27 (40.9%)	0.364
	CR	38 (62.3%)	28 (42.4%)	0.033

TAC, tacrolimus; R, remission; PR, partial remission; CR, complete remission.

#### 3.2.3 Remission time

**FIGURE 3:**
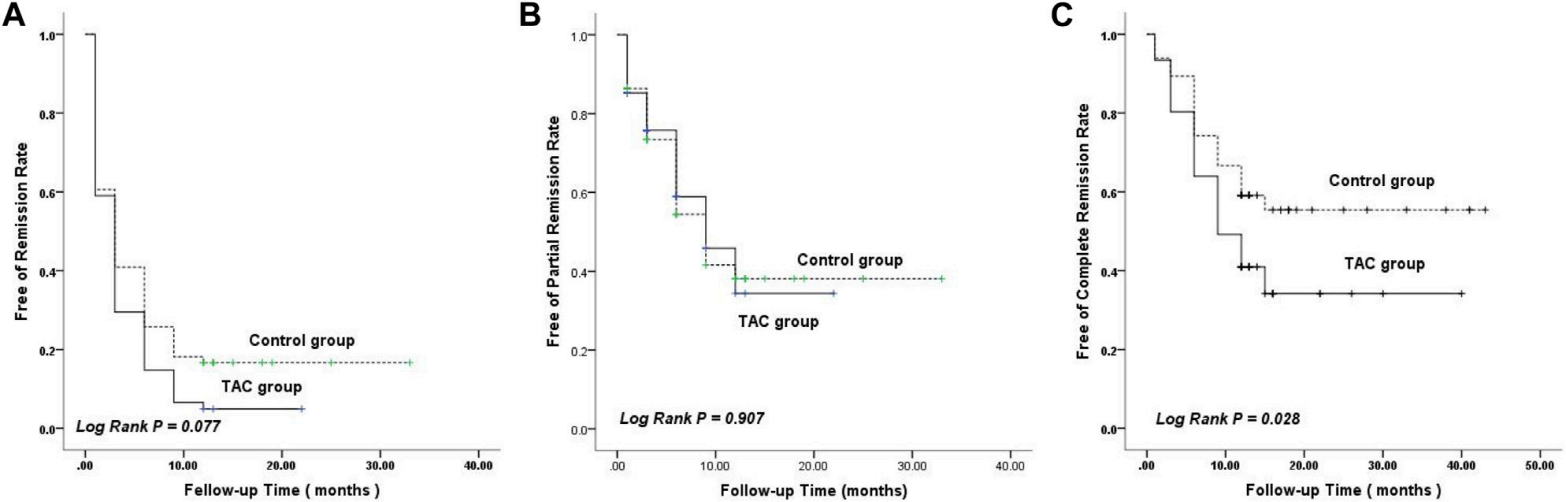
Kaplan–Meier curves ofremission rate. **(A)** Remission. **(B)** Partial remission. **(C)** Complete remission. TAC, tacrolimus.

Based on the Kaplan–Meier curve of remission time, the estimated mean time to achieve remission was 4.20 months (95% confidence interval [CI]: 2.978–5.415) in the TAC group, which was shorter than the mean time of 8.26 months (95% CI: 5.515–11.000) in the non-TAC group; however, the difference was not statistically significant (*p* = 0.077; Figure 3A). Meanwhile, the estimated mean time to achieve PR in the TAC group was similar to that in the non-TAC group (*p* = 0.907; Figure 3B). Notably, the estimated mean time to achieve CR was 18.44 months (95% CI: 14.157–22.723) in the TAC group, which was significantly shorter than the mean time of 27.07 months (95% CI: 22.484–31.660) in non-TAC group (*p* = 0.028; Figure 3C).

### 3.3 Adverse events

**FIGURE 4:**
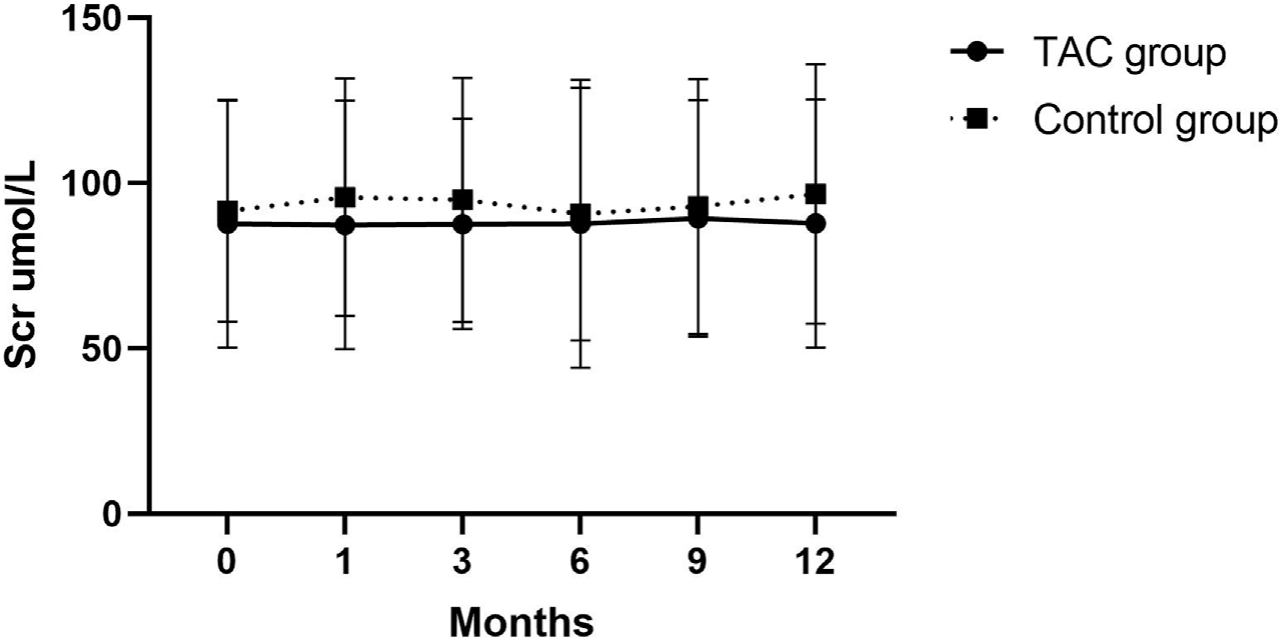
Serum creatinine levels inpatients during follow-up. TAC, tacrolimus.

No severe infectious events occurred during the follow-up period. Main adverse events were abnormal liver function, acute kidney injury, hyperuricemia, elevated blood glucose levels, GC-related osteoporosis, gastrointestinal symptoms, and anemia (Table 4). In addition to GC-related osteoarthritis, the incidence rates of other adverse events were not significantly different between the two groups. Incidence rate of GC-related osteoarthritis (defined as prednisone ≥7.5 mg for >3 months and bone pain) in the TAC group was significantly lower than that in the non-TAC group (9.8% vs. 28.8%; *p* = 0.007). Total prednisone dosage was 269, 895 mg administered to 28 (45.9%) patients in the TAC group and 473,167 mg administered to 52 (78.8%) patients in the non-TAC group. Changes in serum creatinine levels were also analyzed during the follow-up period and no significant differences were observed between the two groups (Figure 4).

**TABLE 4:** Adverse events in the two groups.

Adverse events	TAC group (*n* = 61)	Non-TAC group (*n* = 66)	*p*-value
Abnormal liver function	6 (9.8%)	9 (13.6%)	0.589
Acute kidney injury	1 (1.6%)	2 (3.0%)	1
Hyperuricemia	25 (39.3%)	27 (40.9%)	0.857
Abnormal blood glucose	5 (8.2%)	6 (9.1%)	0.858
Osteoporosis	6 (9.8%)	19 (28.8%)	0.007
Gastrointestinal symptoms	10 (16.4%)	11 (16.7%)	0.967
Anemia	6 (9.8%)	4 (6.1%)	0.519

TAC, tacrolimus.

## 4 Discussion

In this study, we found that TAC effectively reduces proteinuria in patients with non-rapidly progressive IgAN, with a more rapid decline in proteinuria than that observed in the non-TAC group at 3, 9, and 12 months after the onset of treatment. Total remission (95.1%) and CR (62.3%) rates were significantly higher in the TAC group than in the non-TAC group. These results were also observed 9 and 12 months after the onset of treatment. Estimated mean time to achieve CR was 18.44 months in the TAC group, which was significantly shorter than the mean time of 27.07 months in the non-TAC group. In addition, the TAC group did not show an increase in the occurrence of adverse events. These results demonstrate the safety and efficacy of TAC treatment in patients with non-rapidly progressive IgAN. To the best of our knowledge, our study has the largest sample size to date for TAC treatment of IgAN.

Anti-proteinuric effects of TAC in IgAN have been reported in various studies (Hu et al., 2018; Yan et al., 2022), and their findings are consistent with those of our study. For example, TAC was reported to effectively decrease proteinuria in a prospective cohort study of 50 patients with IgAN (24 hUTP ≥ 2.0 g, estimated GFR ≥50 mL/min/1.73 m^2^) (Yan et al., 2022) and a retrospective observational study of 34 patients with refractory IgAN (Hu et al., 2018). A network meta-analysis revealed that TAC improved the remission of proteinuria (RR = 3.67; 95% CI = 1.06–12.63) in patients with IgAN better than that in patients receiving supportive care alone (Han et al., 2020), and the proteinuria remission rate of TAC treatment showed no significant differences (*p* = 0.7) with full-dose GCs (Yan et al., 2022). In our study, TAC treatment led to a significantly higher decrease in proteinuria at 3, 9 and 12 months and significantly higher total remission and CR rates than non-TAC treatment (including immunosuppressive agents CTX and MMF), which has not been reported in previous studies. In our study, the estimated mean time to achieve remission was 4.20 months (95% CI = 2.978–5.415) in the TAC group, which was longer than the mean remission time of 7.0 ± 4.7 weeks in the study of Hu et al. (Hu et al., 2018).

Yan et al. reported an increase in serum creatinine levels after TAC treatment, which later decreased to normal levels when the use of TAC was stopped, indicating a high incidence of renal insufficiency with TAC treatment (Yan et al., 2022). This finding is inconsistent with our results. We found no significant changes in the serum creatinine levels of patients during the follow-up period (12 months) (Figure 4), and acute kidney injury was observed in only one patient (1.6%), which was not significantly different from that in the non-TAC group (3.0%). Moreover, the anti-proteinuric effect and safety of TAC were evaluated based on a smaller sample size in these previous studies, whereas a larger sample size was included in our study. Therefore, the results of this study are more reliable. However, the anti-proteinuric effect of TAC can be reversed after stopping TAC use for 3 months (Yu et al., 2017; Han et al., 2020), indicating the necessity of investigating the long-term effects of TAC in future studies.

We also found that TAC significantly reduced the need for GCs. Only 45.9% of the patients in the TAC group received GCs compared to 78.8% in the non-TAC group. In TESTING studies (Effect of Oral Methylprednisolone on Clinical Outcomesin Patients with IgA Nephropathy: The TESTING Randomized Clinical Trial), GC-related side effects cannot be ignored, although GCs can restore the IgAN uroprotein levels. Our retrospective study found that TAC not only significantly reduced urinary protein levels but also significantly reduced GC consumption, thereby reducing GC-related adverse effects, such as osteoporosis.

Increasing evidence has demonstrated that the mechanism by which TAC reduces proteinuria in patients with IgAN is multifactorial. The first is an immunosuppressive mechanism, in which TAC inhibits the immune response by suppressing the transcription factors involved in activated T cell activity. However, other immunosuppressive agents, including CTX and MMF, were administered to the control group. TAC group showed a more rapid decline in proteinuria than non-TAC group at 9 months (76.9% ± 21.4% vs. 58.2% ± 37.1%; *p* = 0.001) and 12 months (73.2% ± 35.2% vs. 55.4% ± 47.2%; *p* = 0.018). Therefore, other non-immunosuppressive mechanisms may be involved in this process. TAC can induce renal vasoconstriction and increase renal vascular resistance (Textor et al., 1993; Textor et al., 1995); such changes in hemodynamics can decrease the permeability of proteins. This can be confirmed by the findings of a previous study, in which TAC was found to inhibit the production of vascular permeability factor (VPF) derived from T lymphocytes, which is considered the leading cause of massive proteinuria in minimal change nephrotic syndrome (Maruyama et al., 1994). Additionally, an effect of TAC on podocyte cytoskeletal stabilization has been proposed (Zhang et al., 2012). Qi et al. revealed that TAC protects podocytes from injury by upregulating the expression of podocin and nephrin and inhibiting the activation of macrophages (Qi et al., 2016). TAC can inhibit the redistribution of calcineurin and nephrin at the slit diaphragm, which is a unique cell–cell junction of podocytes that functions as an important barrier preventing the plasma proteins from leaking into urine (Wakamatsu et al., 2016). In addition, TAC can decrease proteinuria and restore podocyte injury by inhibiting the expression of angiopoietin-like-4 (a marker for predicting early podocyte injury) (Peng et al., 2014; Li et al., 2015) and calcineurin-binding protein 1 (Wen et al., 2016). Another study revealed that decreased expression of transient receptor potential canonical channels and calcineurin may be involved in the therapeutic effect of TAC on IgAN (Wei et al., 2017).

This study has several limitations: 1) The inherent limitations of the retrospective study design and small sample size, 2) the treatment protocol and doses were not standardized across patients in each group, which maybe confounding factors affecting the results, and 3) renal function and long-term effects of TAC were not investigated. Therefore, a well-designed, randomized controlled study with a larger sample size and long follow-up period is necessary to validate our findings.

In conclusion, TAC accelerated proteinuria remission in patients with non-rapidly progressive IgAN with no increased risk of adverse events. Moreover, TAC decreased the levels of GCs, thereby reducing GC-related side effects.

## Data Availability

The original contributions presented in the study are included in the article/Supplementary Materials, further inquiries can be directed to the corresponding author.
